# What can we learn from the online learning experiences of medical students in Poland during the SARS-CoV-2 pandemic?

**DOI:** 10.1186/s12909-021-02884-5

**Published:** 2021-08-26

**Authors:** Anna Pokryszko-Dragan, Karol Marschollek, Marta Nowakowska-Kotas, Gillian Aitken

**Affiliations:** 1 Department of Neurology, Wroclaw Medical University, Borowska 213, 50-556 Wroclaw, Poland; 2 University Clinical Hospital, Wroclaw, Poland; 3 Edinburgh Medical School, Medical Education, University of Edinburgh, Edinburgh, UK

**Keywords:** Medical education, Undergraduates, Online teaching, SARS-COV-2 pandemic

## Abstract

**Background:**

In March 2020, due to the SARS-CoV-2 pandemic, the Polish government ordered the closing of all medical faculties, with an obligatory shift to online learning. This lockdown continued, with a short period of blended learning, over the time of summer 2020. Distance learning had previously been rarely used within Polish medical schools, so such a sudden transfer was a major challenge. The aim of the study was to explore undergraduates’ perception of online teaching provided at Polish medical faculties during the pandemic and to analyze how these experiences may inform future curriculum development.

**Methods:**

The online survey was addressed to undergraduates at Polish medical faculties in November 2020. The questions captured demographics, epidemiological data and students’ perception of various aspects of online teaching. Responses were subjected to thematic analysis and their distribution compared considering demographic parameters .

**Results:**

Six hundred twenty students from thirteen medical faculties responded to the survey. Major benefits from online teaching perceived by respondents included increased convenience, enhanced quality, a sense of comfort and safety. Major complaints were associated with unsatisfactory content, technical issues, difficulties engaging, poor organization and lack of social life. Students claimed that online teaching required more self-directed learning and discipline and 57.9% considered this impact as negative. 44.5% of respondents took part in educational online activities beyond their scheduled classes. For 49.2% online examinations were reported as more stressful and for 24.8% - less stressful than traditional ones. Differences in the opinions on online teaching were found between men and women, students in early and senior years, Polish and non-Polish ones.

**Conclusions:**

The sudden move online inevitably was problematic for students. Their perspective afforded us the opportunity to consider shortcomings of pre-pandemic undergraduate curriculum. Online education requires a more self-directed learning, which was challenging for many students, so further enhancement of more autonomous study skills seems necessary. Distress expressed by students indicates the need for urgent support with mental health issues.

**Supplementary Information:**

The online version contains supplementary material available at 10.1186/s12909-021-02884-5.

## Background

The rapidly spreading incidence of SARS-CoV-2 (Covid-19) disease, declared as the pandemic by WHO in March 2020, had a thorough and disruptive impact upon medical education in many countries. University hospitals and medical teaching centers had to face unprecedent challenges, prioritizing their main responsibilities: providing medical service, ensuring safety for patients, staff and students and yet continuing to teach within new stay-at-home guidelines. Meeting students’ educational needs and minimizing possible risks, saw undergraduate teaching - with different approaches worldwide - mostly transferred online [1, 2]..

In March 2020, the Polish government ordered the closing of all educational institutions, including universities, with an obligatory shift to online learning. This lockdown affected c. 45,000 undergraduates studying at 22 Polish medical faculties, including c. 11,000 students of so-called English Divisions. These Divisions (organized at the majority of medical faculties) provide parallel curricula in English for non-Polish students, alongside teaching in Polish. This group of international students faced particular difficulties due to the pandemic travel restrictions. “Fast track” of graduation was considered for the final year students, to allow them to join the clinical workforce early in the clinical settings. Furthermore, medical students of all years could volunteer in supervised activities aimed at fighting the effects of the pandemic in various health care or social care institutions.

The educational lockdown at medical faculties was extended till summer 2020, with all the exams held online. At the onset of the new academic year in October, online teaching at most medical faculties was maintained in part-time/blended mode. However, in November due to rapidly increasing rates of SARS-CoV-2 infections and mortality, the restrictions were resumed and on-site teaching was suspended again (with some exceptions, e.g. for final year students).

Worldwide the pandemic challenged existing models of medical education, effectively halting the practice-based teaching that is the backbone of clinical education [3], with students confined to their accommodation to prevent any unnecessary spread of disease [4] but, as noted by Rose [5] this situation cannot continue due to the urgent need to educate the next generation of health care workers. The commentary by Tolsgaard et al. [2] stresses that context matters and it cannot be assumed that what works in one country will work in another. We offer these experiences of the situation in Poland to add to the growing understanding of what has worked well and not so well, in undergraduate medical education as result of the rapid changes instigated in response to the pandemic.

Before the pandemic, distance learning was scarcely used within Polish medical schools, especially in undergraduate curricula, so there was very little institutional knowledge in this area to draw on. A sudden and massive transfer from traditional teaching to an exclusively online setup was therefore a major challenge for both teachers and students. In common with similar experiences from other countries, it was also suggested that these enforced circumstances might become an opportunity to re-assess medical education, develop new innovations and accelerate their use [6–9].

We considered it essential that the students’ perspectives and attitudes towards online teaching during the pandemic were captured to evaluate the current situation in medical education and draw conclusions for further developments in this field. The lessons learned will reveal any shortcomings which need to be addressed, but importantly capture the innovations worth further implementation and development.

The aim of the study was to explore undergraduates’ perception of online teaching provided at Polish medical faculties during the SARS-CoV-2 pandemic, and to analyze how these experiences can inform future curriculum development.

## Methods

The study was based on an online survey, conducted in November 2020.

The questionnaire was developed by the team of authors experienced in clinical education, with the participation of a senior student representative. The questions captured demographic data, exposure to SARS-CoV-2 and students’ perception of various aspects of online teaching. Dichotomous (yes/no) closed items, multiple choice items and open-ended questions were introduced (Additional files 1). The use of open-ended questions allows the collection of rich and unconstrained perceptions of the participants, expressed in their own words. While this meant that participation in the project would take more time and thought than closed questions [10], the research team were particularly interested to capture student’s thoughts in their own words as opposed to merely confirming the preconceptions about the teaching. The questionnaire was created in Google Forms and distributed through social media platforms and websites of medical students’ societies and organizations throughout Poland. The introduction to the questionnaire clearly indicated medical undergraduates as invited participants, provided brief information about the study and contained a consent form. A version in English was addressed to students of English Divisions (international group of students, attending parallel curriculum in English, provided by the majority of Polish medical faculties).

The responses were downloaded from Google Forms and transferred into Microsoft Excel spread sheet. Demographics, epidemiological data and answers to closed items were analyzed quantitatively. The data were thematically analyzed using the process described by Braun and Clarke [11]: initial coding was undertaken independently by two authors (APD and KM), there was good agreement, so the resulting codes were combined and used to identify emerging themes and generate an initial coding framework. Codes were then compared collectively (by all researchers), and iteratively refined as data coding was finalized. All comments were captured within the coding framework, with a high degree of commonality between students’ comments, leading us to believe we achieved data saturation. Thematic analysis is useful in organizing and analyzing large and intricate datasets, however it is also intimately entwined with the experiences and perceptions of those undertaking the analysis. There was a diversity of perspective within the researchers’ team: APD and MNK are experienced neurologists and academic teachers, who have been intimately involved in the running of undergraduate medical programs in Poland (also during the pandemic), KM is a physician in early stage of training, not involved in teaching or undergraduate learning during the pandemic, but with some experience in conducting surveys on health-related issues; and GA, who is an academic experienced in clinical education and online teaching but without any involvement in Polish educational system, provided a degree of externality to both the data collection and analysis.

Distribution of the responses in particular categories was compared between men and women, Polish and non-Polish (English Division) undergraduates, students from preclinical (I-III) and clinical years (IV-VI) of medical studies.

A univariate analysis was performed using STATISTICA (StatSoft Inc., Tulsa, USA; version 13.0). Significance level was established as α = 0.05. Categorical variables were presented as a number and percentage and continuous ones as the mean and standard deviation (SD) or as the median. Chi-squared test for independence was performed to establish significance differences.

The project of the study was approved by Local Bioethical Committee at Wroclaw Medical University (Ref No KB-498/2020). Participation in the survey was voluntary and anonymous; except for basic demographics no data were obtained which would allow identification of respondents. Confirmation of informed consent to participate in the study, provided in the initial part of the questionnaire, was necessary to proceed with responding to the questions.

## Results

### Demographic and epidemiological data

620 medical undergraduates from 13 faculties (all at the major public/state universities) responded to the survey. Table 1 presents demographic structure of the group, which was broadly representative of medical students in Poland.

**Table 1:** Demographic data of the respondents

	Sex			Mean age (years)	Year of studies: n (%)
	Females (n,%)	Males (n,%)	Other		
**Polish Division students** **(*n*** **= 480)**	325 (67.7%)	154 (32.1%)	1 (0.2%)	21.36 (SD 1.83, range: 18-30)	**1**: 90 (18.8%) **2:** 128 (26.7%) **3:** 141 (29.4%) **4:** 72 (15%) **5:** 19 (3.9%) **6:** 26 (5.4%) **Graduates**: 4 (0.8%)
**English Division students** **(*n*** **= 140)**	98 (70%)	42 (30%)		22.51 (SD 2.45, range 16-29)	**1:** 18 (12.9%) **2:** 34 (24.3%) **3:** 20 (14.3%) **4:** 19 (13.5%) **5:** 37 (26.4%) **6:** 12 (8.6%)
**Total (*n*** **= 620)**	423 (68.2%)	196 (31.6%)	1 (0.2%)	21.62 (SD 2.04, range 16-30)	**1:** 108 (17.4%) **2:** 162 (26.1%) **3:** 161 (26%) **4:** 91 (14.7%) **5:** 56 (9%) **6:** 38 (6.1%) **Graduates:** 4 (0.7%)

284 (45.8%) respondents were tested for SARS-CoV-2 presence and 74 (11.9%) were found positive; 187 (30.2%) had undergone quarantine, 282 (45.5%) claimed that a family member or close person was infected or in quarantine. 71 (11.4%) undergraduates volunteered in activities fighting the burden of the pandemic: at hospitals, outpatient clinics and laboratories (assisting doctors, nurses and laboratory technicians; epidemiological check-in at the entrance; data entry); producing and distributing masks and face shields; supplying elderly or quarantined persons with necessary products.

### Undergraduates’ perception of online teaching

137 (22.1%) students had participated in some kind of online learning before the pandemic, in 61 cases - provided by their medical faculty, in 6 - by other medical institutions or organizations, and in 70 - in other field than medicine (school subjects, language courses, other skills).

96.1% (596) of respondents had sufficient access to internet connection and the necessary equipment to participate regularly in online classes.

The themes identified were broadly split between those that were positive about online learning and those that were not.

**Fig. 1:**
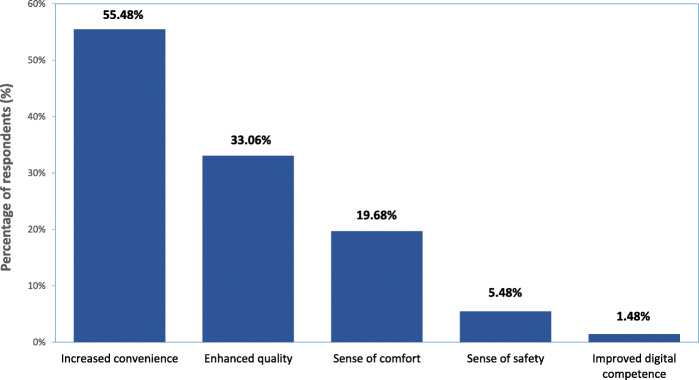
Major benefits of online teaching perceived by respondents to the survey

Several positive aspects of studying online were identified, mainly related to issues of convenience and safety. The major benefits of online teaching as perceived by this group of students included (Fig. 1, Table 2):

- **increased convenience**: saving time (and money) previously spent on commuting and wasted because of inconvenient timetabling (long pauses between the classes, their localization in distant placements); opportunity to take part in the classes independent from circumstances (e.g. staying abroad or during a quarantine); the possibility to organize a more flexible personal study schedule, the flexibility of using pre-recorded or shared materials and planning long-term tasks; a perception of better balance between studying and private/family life;
- **enhanced pedagogical quality**: updated materials in accessible formats (videos, tutorials), individual audio/video perception better than in a crowded or old-fashioned lecture halls, more efficiently used time during the webinar; wider extent of shared or recommended resources; better availability of optional lectures (which had been previously sometimes overlapping or occurred at late hours)
- **a sense of comfort**: staying in familiar surrounds, in comfortable clothes, without pressure from the faculty setting; a chance for better studying/life balance (more sleep, regular meals, enough time for exercise or household duties)
- **a sense of safety** - avoiding contacts and potential exposure to infection
- **improvement in computer competence** (use of different options of communication platforms, preparing documents, presentations or databases)

**Table 2:** Excerpts from the responses to the survey

**Which were in your opinion the major benefits of online teaching?**	
Convenience	• No need to waste time on commuting • Simple to organize, no need to go through whole city for another event • You can participate in class from anywhere. • More flexible about my time schedule • It’s easier to take notes and deal with time management • Learning materials can be shared in an easy and fast way
Enhanced quality	• Shared presentations were really good • Great benefit from pre-recorded lectures - potential to re-watch and re-learn. • Was able to view the contents better online than trying to get a glance at the projector screens during lectures. • It’s easier for me to understand the lecturer online than in a lecture hall. • I enjoyed most live broadcasts from the clinics or an operation room
Comfort	• We can sleep longer and be more focused • Ability to enter the webinar in my own room, get in my favorite robe, make coffee and learn new things • We can attend lectures without leaving home and stay in our comfortable space
Safety	• Lower risk of exposure to Covid; it is safer for us, our families and patients • We can avoid contacts with people in lecture halls or in public transport
Computer competence	• I could improve my computer skills, preparing presentations or graphs • I feel more confident with internet platforms or other digital tools
**What were the major problems which you have experienced during online teaching?**	
Unsatisfactory content	• Lack of contact with patient and lack of learning practical skills. • Practical aspects of subjects will never be the same on a video compared to real life. Also lack of discussion with peers during classes. • Monotonous presentations, not involving students, little chance for active participation • The interaction between teachers and students is greatly impaired and less personal. It is more difficult to ask questions and get feedback because you can’t see them in person.
Technical issues	• Weak internet connection • Technical difficulties on both sides i.e. student and teacher • Problems with platforms - unstable connection, server failures or limited number of participants allowed.
Difficulties engaging	• No motivation, I find classes boring and not enjoyable. • Long/disorganized days, difficulty concentrating, lots of screen time. • Don’t learn as much as if it would be in person. This all have lead to lack of motivation
Lack of social interactions	• Personal contact with other students missing. I think that’s very important. And for me a huge motivator. • The social aspect of seeing and interacting with your colleagues is missing, which is very important and can be detrimental to mental health
Poor institutional organization	• The departments use different tools even though one platform has been picked to be the main one. It takes a lot of time to send every homework and report etc. Materials are widespread through mails, websites ... difficult to find them and put together. • Sometimes meetings are longer than they are scheduled or lectures are postponed. The information about schedule not always updated on time. • The overload of the material that we have to learn. There is much more material to manage before the test - and much less time for that
**Has online teaching influenced your own studying? If yes, in which manner?**	
• I have to spend a lot more time to teach myself something, it has practically become self teaching at this point. • I find it harder to concentrate, more easily distracted. It got me out of my regular study routine, which is really hard. • I’m less motivated and feel left alone with the stuff we have to learn without any advice or feedback. • It demands from me more discipline • I spend more time studying for the subjects that particularly interest me (more energy to do so, less tired than after normal classes) • I moved digital - make notes in computer and look up a lot of resources online	

30 respondents (4.8%) identified no positive aspects of online education (answering “none” or leaving a blank space).

**Fig. 2:**
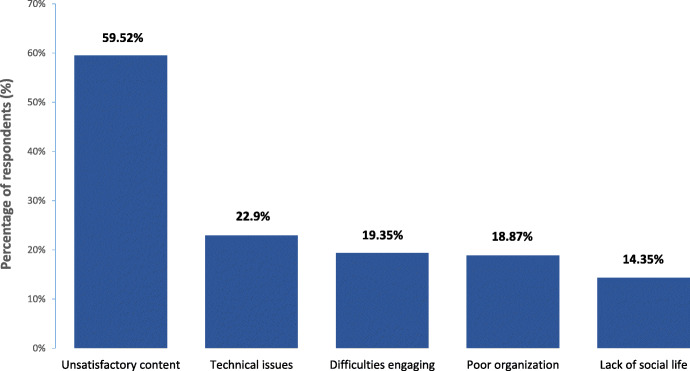
Major complaints associated with online teaching reported by respondents to the survey

The situation was not all positive. Major complaints associated with online teaching were also reported (Fig. 2, Table 2):

- **unsatisfactory content**: the difficulties of teaching practical skills online (bedside and laboratory classes), poor quality of some classes - monotonous, time consuming - difficult to keep focused; few opportunities of face-to-face interaction with teachers and peers
- **technical issues**: poor internet connection, failures of overloaded servers and communication platforms; teachers unable to manage the problems or demands of online mode; need to compromise home-office or online learning of other family members;
- **difficulties engaging:** problems with concentration, loss of motivation, emerging stress and frustration
- **poor institutional organization**: lack of clear structure and consistency within university/faculty, frequent changes in schedule, lack of coordination between particular departments - conflicting timetables, various platforms used with resources widespread across these; overload with material with no adjustment of syllabus to exceptional situation; problems with completion of credits; poor communication between faculty staff and students
- **lack of social life**: no contacts with peers - feeling of isolation affecting mental health, poor integration of 1st year students, lack of discussions and common projects helpful in learning

18 (2.9%) respondents identified no negative aspects of online education (answering “none” or leaving a blank space).

Students offered examples of teaching formats which they found to be particularly valuable, these included:

- recorded lectures and webinars, followed by “questions & answers” session, available for multiple playback
- interactive clinical case-based discussions in small groups
- workshops with interactive tasks and quizzes (e.g. labeling relevant findings, completion of schemes, filling the gaps)
- tutorials and films illustrating experiments in basic sciences and clinical procedures (e.g. neurological examination, endoscopy, surgical interventions)
- “live streaming” from the operation room, ward round or outpatient clinic
- online interviews with real or simulated patients

Overall, the respondents claimed that online education, in comparison with traditional teaching, required more self-directed learning and greater discipline in the organization of learning requiring a high degree of intrinsic motivation. The majority of students (57.9%) perceived this impact as negative: they found it more difficult to focus on particular problems and retain information, were getting more fatigued with constant exposure to their computer, felt less motivated to learn regularly without direct contact with teachers and traditional assessment forms, suggesting that this had a negative impact on their learning (Table 2). However, a smaller percentage of students (18%) noticed positive influences of this period upon their learning. Some of them adapted new techniques of learning (made more informative notes, “moved digital” so improving their computer skills). Others reported becoming better organized in their scheduling of learning according to their personal needs, felt encouraged to look for additional resources and developed interest in specific topics or specialties (Table 2). Apart from obligatory classes, 276 (44.5%) undergraduates took part in other educational online activities and used online resources, provided by students’ organizations, medical journals, scientific and clinical specialties societies.

When asked about the faculty attitude to teaching, 314 (50.6%) respondents felt that teachers tried to provide some kind of substantial and emotional support in this exceptional situation. 170 (27.4%) students contacted their faculty because of problems associated with online teaching (technical issues, course and content of classes, formal issues) and in 107 (62.9%) cases they obtained appropriate assistance.

Online examinations taken by respondents were mostly tests and other written forms (98.2 and 41.6%, respectively), and 54.7% had undergone oral examinations. For 305 (49.2%) of students’ online examinations were reported as more stressful than traditional ones, for 154 (24.8%) less stressful and 157 (25.3%) reported no change in their perception of degree of stress.

The main sources of stress related to anxiety associated with unpredictable internet or server failure resulting in interrupted/failed examination and time pressure during the tests (limited and unified time for each answer, one-way navigation mode without ability to return to previous questions, problems with quick typing). Some students were afraid to be wrongly accused of cheating or found the results of examination unfair related to concerns of cheating by other students. Faculty arrangements to prevent cheating or minimize its impact (obligatory sharing cameras, recording oral examination sessions, increased degree of test difficulty and/or pass threshold) were also perceived as stressful. Fear about the new and unusual form of examinations was also increased by lack of clear instructions or changing rules without appropriate information from the faculty.

Those who found online examinations less stressful than traditional ones, felt comfortable in more informal settings, reporting being better rested and fed prior to the examination, which resulted in feeling more relaxed. More comfortable seating and background music was felt to reduce the pressure perceived in an examination hall, away from the presence of supervising teachers and other stressed students. They also appreciated the possibility of using notes or other resources, allowed during some examinations, although it should be noted that this does not only relate to online examinations.

**Fig. 3:**
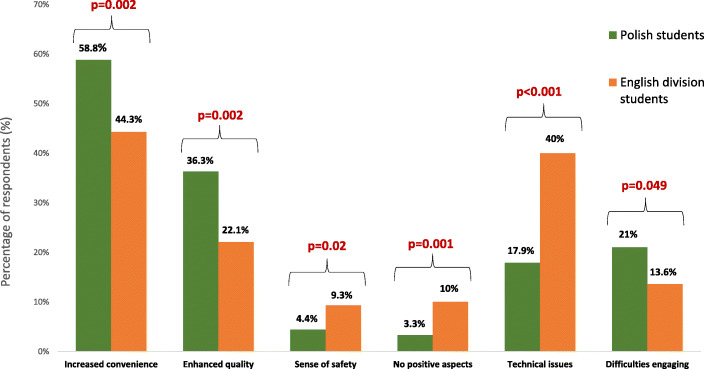
Significant differences in perception of online teaching between Polish and English Division students. *P* values of χ^2^ tests are presented for each comparison

There were some marked differences between Polish and English Division students (Fig. 3). Polish students in comparison with those in the English Division more often appreciated the convenience and enhanced quality of online teaching. They were also more likely to complain about motivation/concentration loss but were less likely to complain about technical issues; were less concerned about avoiding risk of infection and less frequent declared that online teaching had no positive aspects.

**Fig. 4:**
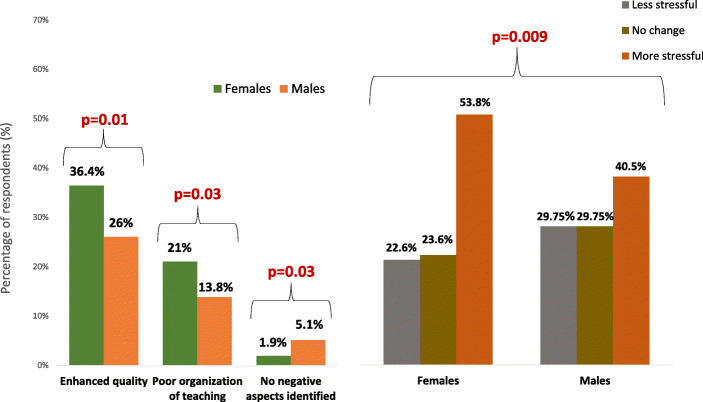
Significant differences in perception of online teaching and online examinations between females and males. P values of χ^2^ tests are presented for each comparison

Female undergraduates in comparison with men more often appreciated enhanced quality of webinars and were more often disapproving of poor organization of teaching, less frequent declared that online teaching has no negative aspects and more often found online examinations more stressful that traditional ones (Fig. 4).

**Fig. 5:**
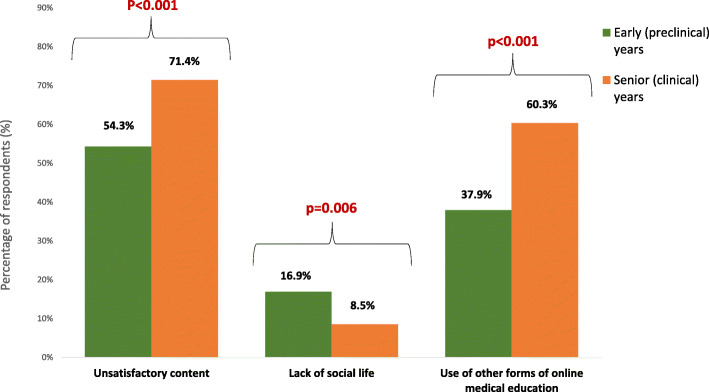
Significant differences in perception of online teaching between students from early (preclinical) and senior (clinical) years. P values of χ^2^ tests are presented for each comparison

Students from senior/clinical years were more dissatisfied with content of classes and less concerned with lack of social interactions; they more often used other forms of online education than early years students (Fig. 5). No doubt associated with their impending graduation.

English Division students (20% vs 9%, *p* < 0.001) and those from clinical years (16.4% vs 9.3%, *p* = 0.01) were more often engaged into voluntary activity against the pandemic.

No other significant differences were found on comparative analysis of responses from the above subgroups of participants.

## Discussion

Lack of experience in remote forms of medical education in Poland was probably one of the major factors influencing shortcomings in organization and quality of teaching, and the resultant students’ approach to learning. Internet-based resources and tools for remote teaching, already existing in some countries [6, 12, 13], were not available for easy adaptation to continue education during lockdown of medical schools in Poland. Previous experiences with online modes of learning were shown to affect students’ remote learning proficiency and their satisfaction with education during the pandemic positively [9, 14, 15].. It is logical to assume that this situation is similar for staff tasked with moving to online teaching. A lack of institutional knowledge in online teaching throughout Polish medical schools inevitably made this transition more challenging than in countries with better established learning technologies.

Undergraduates’ attitude towards online teaching was affected by the stage of their studies. Senior students were more dissatisfied with the content of classes, which would not allow them to acquire competence in clinical subjects, in particular the practical skills needed for successful clinical practice. Probably because of this dissatisfaction they searched for other forms of online education, choosing from a wide range of webinars in clinical specialties, which were freely offered during the pandemic. Nevertheless, they claimed that even extended virtual teaching could not replace bedside classes. This mirrors the experiences of senior students responding to similar surveys in other countries, who were also less satisfied with online teaching and expressed their concern about inappropriate preparation for clinical rotations and ultimately for transition from undergraduate to physician [6, 13–19]. For early years students, online delivery was more acceptable in gaining theoretical knowledge, but they missed interactions with teachers and peers and the overall opportunity to integrate with students’ community at the beginning of their university experience. English Division students, who mostly stayed in their native countries, appreciated remote access to classes and sense of safety, but they valued convenience and enhanced quality of online teaching less than their Polish classmates. They more frequently experienced technical problems and reported language barriers (for the majority of students in the division do not have English as their native language) that prevented them from effective participation in online classes or communication with the faculty to solve emerging problems. All these aspects need to be addressed and possibly compensated upon return to less socially-distanced teaching - e.g. with more effective time management with training focused on clinical skills, encouraging better engagement with optimal use of interaction and feedback, as well as improvement in communication on individual and institutional level [9, 20–22].

Another important issue which arises from the students’ responses, is associated with distress and mental health. Almost 50% of respondents found online examinations more stressful than standard ones, for a variety of reasons. Apart from that, many students spontaneously expressed being distressed and frustrated while identifying shortcomings of online teaching or its impact upon their individual learning. They indicated extrinsic (poor organization, technical problems) and intrinsic (difficulties engaging, loss of motivation, little capability of self-directed studying) factors contributing to their distress. Furthermore, lack of social contacts and isolation were declared to badly affect mental well-being. Other studies in this field also showed a high level of anxiety and distress among medical undergraduates, expressed in the open comments [12, 17, 19, 23, 24] or evaluated with psychometric scales [25, 26], and correlated with their learning context and lifestyle habits. In these extraordinary conditions a need for pastoral and tutorial support from teachers should be highlighted, to improve academic performance as well as mental well-being [20, 21]. In this regard, half of the respondents felt that teachers tried to provide some kind of support. Considering the expected increase in mental health problems as a result of pressure from various consequences of the pandemic, an appropriate system of professional counseling and treatment should be developed and strengthen by medical faculties. Both educators and students would need support in their ability to recognize symptoms of anxiety, depression and distress, as well as to develop effective coping strategies. Psychological care should be not only formally available, but also normalized and destigmatized, encouraging especially undergraduates to apply for necessary counseling [25, 26].

Students’ perception of online education also offered insight into drawbacks of pre-pandemic curricula [12, 27]. Any structural problems within medical schools have been exposed by the current situation (ineffective organization, poor communication between students and faculty) and do not solely relate to online education. The questions asked of this group of students were not discriminatory enough to uncover how much dissatisfaction relates to the move to online teaching and how much to wider organizational concerns. More positive perceptions can be more clearly linked to online learning (accessibility of resources, convenience of timetable) and are important points to consider in future curriculum developments. However curricular reviews need to look beyond the mode of delivery and consider students preparedness for study. The respondents’ remarks about impact of online education on their individual studying revealed their problems with adapting to a more active approach to self-directed learning than they had previously be used to. The participants in other studies also perceived their autonomous learning (without regular guidance from teachers) as ineffective and complained of lack of self-discipline [24, 28, 29]. These findings hint at the need for curriculum to become more student-centered and with a greater focus on teaching study skills at the outset. Wider implementation of educational methods which enhance students’ active participation (e.g. problem based learning, branching scenarios) is also worth considering [8, 30].

Our work highlights several structural problems with undergraduate medical curricula that have been exposed by the pandemic, these compound the existing challenges faced by those designing such curricula. For example, how to accommodate the exponential growth in knowledge to already overfull curricula [31] and how to train doctors best to work in the future of an aging population largely managed in the community setting [31]. It is important that time is taken to consider what lessons the pandemic offers to future iterations of curricula.

Similarly to any other form of teaching, teaching online has both advantages and disadvantages. It can be seen here to be particularly effective in the earlier years of medical curricula where much of the basic sciences are taught. The current delivery was found to be less helpful for senior students preparing for independent practice, a point worthy of consideration by academic administrators when prioritizing limited resources in forthcoming years. Conversely earlier year students reported the lack of social interaction in online teaching more concerning than senior students. To integrate and support these undergraduates, some virtual initiatives might be undertaken, associated with content of teaching (e.g. discussion on learning techniques, promotion of healthy lifestyle) or with other areas (e.g. cultural or sport events), hopefully to be continued within post-pandemic activities of medical schools.

Studies such as this are important in elucidating students’ opinions about digital learning for its successful development [14, 32]. Experiences from this rapid and enforced transition to the use of technology for medical education should be considered for further implementation as a valuable and on-going adjunct to on-site teaching. Webinars with multiple viewing option were reported as interesting and efficient for individual learning [6, 33]. Their post-pandemic use could increase attendance at lectures and encourage interest in a wider range of topics. Live broadcasts, films or virtual case presentations, appreciated by students, might complement bedside classes, especially with regard to limited access to the patients (rare diseases, surgical procedures etc.). The introduction of innovative virtual tools may enhance engagement and interactive participation of learners [8, 25, 30]. However, these initiatives do not come without a cost. Increases in the digital competence of faculty requires further investment, both in time and resource, and is considered mandatory for improving the quality of online teaching. While academic staff are largely responsible for the content of teaching materials, this study also highlights the general dissatisfaction for the organization of online teaching. This requires further investigation to uncover if this merely relates to the difficulties associated with the rapid move to online teaching or represents wider systemic issues.

Other studies published on the topic of moving clinical teaching online are broadly suggestive of good acceptability but also raise issues around the unreliability of existing technology [34, 35]. The concepts of moving teaching online must be recognized as a problematic one. A clinical course is not a static or portable entity that can simply be moved online [21] but is a complex mix of content, experience, activities and learning. Certainly, content can be delivered online but other aspects are more difficult in clinical programs. The authenticity of the clinical learning environment cannot be replicated online where clinicians can role model ‘how’ to be a doctor. This authenticity of the learning environment has been identified as important to students learning in specialties such as general practice [36].

Online teaching has many uses in clinical education but is unlikely to replicate the messy and unpredictable reality of the clinical environment. Educators have done their best under challenging conditions but it is time to reassess what we wish to retain from the near-universal move to online teaching.

### Limitations of the current study

Despite low estimated response rate (1.3%), the findings from this survey may be considered as representative for several reasons. The respondents represented all the major medical faculties (including English Divisions) at public universities, and the demographic structure of the participants corresponded with current data for the wider population of medical undergraduates in Poland.

The previously published study on perception of online teaching addressed to Polish medical students [30] was conducted a few weeks after obligatory shift to remote education and included a range of formulated options to choose from. We believe that the construction of our survey (with predominating open-ended items) and its distribution mode (aiming at preserved anonymity) encouraged expression of sincere opinions. Furthermore, our survey covered various aspects of online teaching and was conducted after a few months of pandemic educational lockdown, so the responses provide the students’ perspective after they had had the opportunity to become familiar with the various online teaching employed.

Limitations of the study are common to all work relying on data collected at one point in time and would benefit from further verification from other researchers. We offer these thoughts to stimulate debate in other areas and learn from others about enforced changes to medical education worldwide. We do not know if those who responded were more of less likely to have had a very good, or very bad experience and plan to undertake some follow-up interviews in the near future to confirm these preliminary results. Participants from other health sciences faculties or multiple responses from the same person, while unlikely, could not be identified or eliminated. We make no judgment on the effectiveness of the online teaching by assessing differences in student attainment to previous year and suggest this is an area for future investigation. This study focused on students’ perception of this specific situation and what lessons can be learned regarding the effectiveness of pandemic medical education. The survey was addressed to students only, so the teachers’ perspective was not considered, which would have provided a more nuanced picture. Further investigation in this field might include the medical teachers’ reflections on online education, as well as confrontation of pandemic and post-pandemic approach to online versus traditional modes of medical teaching.

While the questionnaire was designed to be simple to understand, using clear instruction and was piloted prior to use, the impact of the researchers on the results must be acknowledged. Any qualitative analysis must be considered in terms of its context. We offer a full description of the researchers, our experiences and roles to allow a fuller understanding of our positionality and allow readers to judge the applicability of these findings to their own context.

## Conclusions

Medical undergraduates in Poland noticed both positive and negative aspects of online teaching during the SARS-CoV-2 pandemic and claimed that it demanded more active and self-directed approaches to learning. Perceived substantial and organizational problems mostly resulted from the sudden transfer to online education without previous experience in this field, but also revealed several shortcomings of pre-pandemic curriculum, which need to be addressed. Some forms of digital teaching should be considered for further implementation as an effective adjunct to on-site education. Stress and frustration expressed by undergraduates indicates the pandemic has taken its toll on the mental health of this group of students, which should be urgently addressed.

## Supplementary Information

**Additional file 1.**

## Data Availability

The datasets used and analyzed during the current study are available from the corresponding author on reasonable request.
