# Supplementary material for: What can we learn from the online learning experiences of medical students in Poland during the SARS-CoV-2 pandemic?
Source: BMC Med Educ. 2021 Aug 26;21:450. doi: 10.1186/s12909-021-02884-5 (PMC8390042; doi:10.1186/s12909-021-02884-5)
Supplement: Supplementary file 1 — Additional file 1. [file 12909_2021_2884_MOESM1_ESM.docx]

Additional file 1 : The survey used in the study

Age: Gender:

Name / location of University:

Year of studies:

1. Have you ever participated In online education before the pandemics? YES/NO

If YES, In which field? ..........................................................................................

1. Please mark all the forms of online teaching provided by your medical faculty during the pandemics

- webinar/lecture for large group
- webinar/workshop for small group
- access to text files or presentations
- films
- educational tests, quizes etc.
- other - please specify .........................................................

1. Did you have an access to Internet and the equipment necessary to participate fully in online teaching? YES/NO

1. Which were in your opinion the major benefits of online teaching ?

.....................................................................................................................................................

1. What were the major problems which you have experienced during online teaching?

...........................................................................................................................................................

1. Please give an example of online teaching which you have found especially helpful in learning

(subject/topic and form of classes)

…………………………………………………………………………………………………………………………………………………

1. Apart from realizing the curriculum, do you feel that teachers provided some kind of support In this extraordinary situation? YES/NO
2. Has online teaching influenced your own studying? YES/NO

if YES, please specify, in which manner ..........................................................................................

1. Apart from online teaching provided by your faculty, have you used any other forms of online medical education? YES/NO

If YES, please specify .......................................................................................................................

1. Please mark all the forms of online exams which you took during the pandemics

- single/multiple choice test
- test with open questions
- other form of written exam (case report, essay etc)
- oral exam

1. In comparison with your previous (traditional) exams, did you find an online exam

- more stressful
- less stressful
- no difference

If "more" or "less stressful", please specify briefly …………………………………………………………..

1. Have you contacted appropriate person/service at your faculty because of any problems with online teaching ? YES/NO

if YES, what kind of problem did you report? ....................................................................................

Did you obtain appropriate help/assistance? YES/NO

During the pandemics, did you:

- return to your country of origin?
- stay in Poland?
- stay in another country?

Were you tested for the presence of SARS-COV-2? YES/NO

Were you positive for SARS-COV-2: YES/NO

Did you undergo the quarantine? YES/NO

Did your family member, friend or close person was positive for SARS-COV-2 or undergo the quarantine? YES/NO

Have you participated in voluntary activities fighting the burden of SARS-COV-2 pandemics? YES/NO

If YES, please specify ....................................................................................................................................
